# Rapid and Continuous Modulation of Hippocampal Network State during Exploration of New Places

**DOI:** 10.1371/journal.pone.0073114

**Published:** 2013-09-02

**Authors:** Caleb Kemere, Margaret F. Carr, Mattias P. Karlsson, Loren M. Frank

**Affiliations:** 1 UCSF Center for Integrative Neuroscience and Department of Physiology, University of California San Francisco, San Francisco, California, United States of America; 2 Department of Electrical and Computer Engineering, Rice University, Houston, Texas, United States of America; 3 CNC Program, Stanford University, Palo Alto, California, United States of America; University of Alberta, Canada

## Abstract

Hippocampal information processing is often described as two-state, with a place cell state during movement and a reactivation state during stillness. Relatively little is known about how the network transitions between these different patterns of activity during exploration. Here we show that hippocampal network changes quickly and continuously as animals explore and become familiar with initially novel places. We measured the relationship between moment-by-moment changes in behavior and information flow through hippocampal output area CA1 in rats. We examined local field potential (LFP) patterns, evoked potentials and ensemble spiking and found evidence suggestive of a smooth transition from strong CA3 drive of CA1 activity at low speeds to entorhinal cortical drive of CA1 activity at higher speeds. These changes occurred with changes in behavior on a timescale of less than a second, suggesting a continuous modulation of information processing in the hippocampal circuit as a function of behavioral state.

## Introduction

Hippocampal activity is required for both laying down the memories of ongoing experience and converting these initial, labile traces into long lasting distributed representations [1]. Standard models of hippocampal function have posited two distinct network states associated with these processes [2]. The creation of new memory traces is thought to occur during active exploration when the ∼8 Hz theta rhythm is prominent and hippocampal place cells are active in specific regions of the animal’s environment. During this state, highly processed sensory information from the entorhinal cortex (EC) drives activity throughout the hippocampal circuit [3], [4].

In contrast, the consolidation process that leads to the stabilization of long-term memories is thought to occur during periods of awake stillness or slow-wave sleep. At these times, sharp-wave ripple (SWR) events are prominent [5]. These events generally occur when bursts of activity generated in hippocampal area CA3 propagate through CA1 to distributed neocortical circuits [6]–[10]. In the absence of sensory input, the highly plastic and recurrent CA3 is thought to act as an auto-associative pattern completion network [11] that can reinstate learned patterns. Neural activity during SWRs in awake animals frequently involves replay of stored patterns, associated with past experience [12]–[15].

These two network states are thought to reflect at least in part the strength of the Schaffer Collateral (SC) pathway from CA3 to CA1. Measures of field excitatory post-synaptic potential (EPSP) size in response to commissural stimulation suggest that the effective strength of CA3 input to CA1 is substantially reduced when animals are running as compared to periods of immobility [16], [17]. Thus, strong CA3 input to CA1 is associated with the still/consolidation state while weak CA3 input to CA1 is associated with the moving/encoding state.

While this framework dominates current thinking about hippocampal activity, a number of findings do not fit well with this two state model. In addition to playing a role in memory consolidation, CA3 input to CA1 is important for one-trial learning in a novel context [18], which need not occur during periods of stillness. Furthermore, recent work has identified slow (∼20–50 Hz) and fast (∼50–100 Hz) frequency ranges of the gamma rhythm in CA1 that correspond respectively to CA3 or EC coherence with CA1 [19]. Both ranges of gamma are seen during exploration, suggesting that CA3 influence can be strong during movement. Further, the peak gamma frequency in CA1 has been shown to increase smoothly with increasing speed [20] but the relationship between this observation and weaker CA3 input during movement remains unclear. Finally, during new experiences when the hippocampus is critical for forming memories, animals exhibit complex, exploratory behaviors [21] that may not be captured in our current understanding of a single “moving” state. Indeed, previous work has demonstrated a number of changes in hippocampal activity associated with new experiences, including decreases in theta frequency [22], changes in the preferred theta phase of CA1 spiking activity [23] and increases in coordinated reactivation during SWRs that can be seen when animals are moving as well as when they are still [24], [25]. These findings suggest that particularly during novel exploration, there may be a more nuanced relationship between movement and information processing in the hippocampal network. Here we investigate the dynamics of the CA3-CA1 network during learning to understand how moment-by-moment changes in behavior seen in animals exploring a new place are reflected in the way the hippocampal network processes information.

## Materials and Methods

### Surgical Implantation and Microdrive Specification

#### Recording-only experiments

Neuronal activity was recorded in 7 male Long Evans rats weighing between 400–600 grams with 30 independently movable tetrodes assembled in two bundles targeting dorsal hippocampal region CA1 and dorsal CA3 (n = 4; all coordinates relative to bregma: AP −3.7 mm, ML ±3.7 mm), CA1 and medial EC (n = 2; *CA1* −3.6 mm AP, 2.2 mm ML, *MEC* −9.1 mm AP, 5.6 mm ML at a 10° angle in the sagittal plane), or CA1, CA3 and EC (n = 1). Analyses using data from the CA1-CA3 animals have been reported previously [13], [26]. On the days following surgery, hippocampal tetrodes were advanced to the cell layers until characteristic LFP patterns were observed and entorhinal tetrodes were advanced through primary visual cortex until no visual responses were observed and proper depths were reached. All spiking activity was recorded relative to a reference tetrode located in the corpus callosum. Local field potentials were recorded relative to ground. Tetrode positions were adjusted after daily recording sessions for all tetrodes that had poor unit recordings. Recording was initiated when LFP characteristics (sharp wave polarity, theta modulation) and neural firing patterns indicated that the target regions had been reached. On rare occasions, some tetrodes were moved before recording sessions but never within 4 hours of recording.

#### Stimulation experiments

In a second set of experiments, we measured field potentials following stimulation of CA3 inputs to CA1. We recorded electrically evoked responses in 4 male Long Evans rats weighing between 400–600 grams. Movable 10 kΩ tungsten or platinum iridium bipolar stimulating electrodes (MicroProbes for Life Science, Gaithersburg, MD) were lowered into either the ventral hippocampal commissure (n = 3; −1.3 mm AP, +1.0 ML) or contralateral CA3 to stimulate the Schaffer collateral pathway (−3.6 mm AP, −3.6 mm ML). Either a multi-tetrode array (n = 2; −3.6 mm AP, −2.2 mm ML) or a 16-channel linear array silicon probe (NeuroNexus Technologies, Ann Arbor, MI; n = 2; −3.6 mm AP, −3.6 mm ML or −2.2 mm ML) was implanted above the dorsal hippocampus. The depth of stimulation electrodes was set to maximize evoked responses. For all electrical stimulation experiments the level of current in the 0.2 ms biphasic pulse (A-M Systems, Sequim, WA) was set to evoke easily measurable field post-synaptic potentials (fPSPs) in area CA1, typically halfway between threshold and maximum response, and between 50–250 µA. The level of current was always held constant across multiple sessions recorded in a single day.

All procedures were done according to the recommendations in the Guide for the Care and Use of Laboratory Animals of the National Institutes of Health and approved by the UCSF Institutional Animal Care and Use Committee (Permit Number: AN089144). Surgical procedures were performed using appropriate anesthetic and analgesic agents, and all efforts were made to minimize suffering.

### Neural Data Acquisition

Data were collected using the NSpike data acquisition system (L.M.F. and J. MacArthur, Harvard Instrumentation Design Laboratory). During recording sessions, we recorded both local field potentials (filtered 0.5–400 Hz and sampled at 1500 Hz) and threshold-crossing spike snippets (40 samples at 30 kHz, filtered 300–3000 Hz or 300–6000 Hz). Single neuron data were clustered using custom software (MatClust, M. Karlsson) as described previously [26]. We used standard waveform and mean rate criteria to separate putative excitatory pyramidal cells from putative inhibitory fast spiking cells [27], [28].

### Behavior

#### Recording only experiments

**Figure 1:**
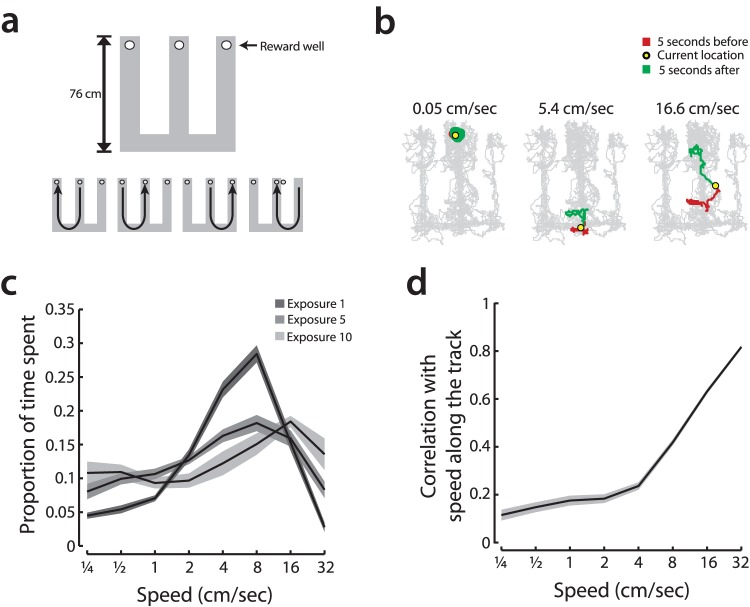
Learning corresponds with a decrease in intermediate-speed exploratory behaviors. **a,** **(top)** Rats were introduced to a novel W shaped environment where they learned to perform a spatial alternation task over multiple 15 minute exposures. **(bottom)** Animals were rewarded at the ends of each arm when they performed the correct sequence of trajectories: from center arm to left to center to right to center… **b,** Rat behavior was monitored using head-mounted infrared diodes tracked with an overhead camera, and smoothed speed was extracted from this data. Three examples of extracted speed also show the surrounding behavioral trajectory over 10 seconds in slow, medium, and fast cases. **c,** The head movement speed distributions for exposures 1, 5, and 10 to an initially novel W-track. Means are shown with solid lines and standard errors with shaded regions. **d,** Correlation between speed, measured in two dimensions, and linear speed along the axis of the track. Lower correlations at intermediate speeds are consistent with exploratory behavior.

Prior to implantation, animals were food deprived to 85–90% of their baseline weight and pre-trained in a separate room to run back and forth on a raised track for liquid food reward (evaporated milk) at each end. Animals were exposed sequentially to two physically different W-shaped environments (76×76 cm with 7 cm wide track sections, Figure 1a) and during intervening sessions in a high walled rest box (20 minute rest periods, floor 25×34 cm; walls, 50 cm tall) [26]. Each rat was exposed to the first initially novel W-track and after either three days (n = 6) or five days (n = 1) of two run sessions per day on a second, now familiar W-track. The two W-tracks were oriented at 90 degrees with respect to one another and were separated by a high barrier to ensure distinct visual cues. Both environments had a reward site at the endpoint of each arm and animals were rewarded for performing a continuous alternation task [26], [29]. Rapid learning of this task requires an intact hippocampus [30]. Throughout the experiment, animals completed two or three 15-min run sessions per day. Data from the more familiar track was used for all analyses except for within day comparisons to study the effect of novelty. For novelty effects, comparisons were made between the first session on the second track and the familiar session immediately preceding it for animals 1, 3, 4, and 5. Animal 6 was not included for within day comparisons due to electrical artifacts in LFP recorded in the novel environment.

#### Stimulation experiments

Prior to implantation, animals were food deprived to 85–90% of their baseline weight and trained to run back and forth on a raised track for liquid food reward (sweetened soy or evaporated milk) at each end. We used two different track designs, a linear track (either straight or U-shaped; 4 animals, 22 epochs) with food wells at each end and a W-shaped maze (described above; 3 animals, 9 epochs). All pre-training was done in the recording room to familiarize animals with both the tracks and the spatial context. We recorded one to three run sessions each day interspersed by 20-min rest periods. To measure synaptic strength as rats behaved, we triggered electrical pulses at 0.05–0.1 Hz for several minutes at a time. Thus, for each animal and day of behavior, we recorded the fPSP response to CA3 stimulation in area CA1 (31 data sets, minimum 46 and maximum 193 pulses).

### Histology

Following the conclusion of the experiments we made microlesions through each electrode tip to mark recording locations (30 µA for 3 sec). After receiving an overdose of Euthasol, animals were perfused intracardially with isotonic sucrose followed by 4% paraformaldyhyde. Brains were frozen and cut either coronally or sagittally at 50 µm sections, and stained with cresyl violet to verify recording locations. Exact reconstructions were not available for one CA1-EC recording animal, so we verified the locations of tetrodes targeted to the EC using theta coherence and theta polarity relative to CA1 as well as the absence of ripples in the LFP at the time of CA1 ripples [31]. EC tetrodes were included for analysis only if the theta and ripple related activity was consistent with layer 3 recordings. We also verified that results from these tetrodes were consistent with results from tetrodes with histologically verified recording locations.

### Behavioral Data Acquisition and Speed Estimation

An infrared light emitting diode array with a large and a small cluster of diodes was attached to the preamps during recording. Following recording, the rat’s position on the track was reconstructed using a semi-automated analysis of digital video of the experiment. The position of the front and back tracking diodes extracted from the video were first smoothed using a nonlinear method [32]. We computed speed by taking the difference in position and then smoothing using a Gaussian kernel with standard deviation 0.5 s and a total length of 6 seconds. As shown in Figure 1b, the resulting measure spans the range of behavioral variation from stillness (e.g., while eating), low speed movement (e.g., uncertainty at a decision point), and fast movement (e.g., while running along an arm towards the food well). Here we note that we used this speed measure because changes in speed could capture a surprising amount of the variability in physiological variables. Thus, although a single variable can never capture the full range of behavioral variability, measuring the two dimensional speed of the animal’s head provided important insights into the dynamics of hippocampal circuits. To calculate speed along the axis of the track (Figure 1c), we first projected two-dimensional trajectory of the rat onto the linear axes of the track [26] and then calculated its first derivative. This measure differs from the speed measure used throughout the rest of the work because it ignores movements made perpendicular to the main axis of the track.

### Spectral Analysis

#### Speed spectrogram (Figure 2)

A windowed power spectrum was computed for a tetrode located in the CA1 cell layer using the multi-taper method from the Chronux toolbox. Multi-taper estimates of the power spectrum were obtained for 0.5-second non-overlapping windows and a z-score was computed for each frequency band. Thus for each 0.5 second bin, we obtained a normalized measure of power for each frequency band in units of standard deviations from the mean. We assigned each of these 0.5 second normalized power spectrum to a logarithmically spaced speed bin and then plotted the mean power for each speed bin. Note that this choice of bin sizes is not appropriate for lower frequency physiological rhythms such as delta or theta.

**Figure 2:**
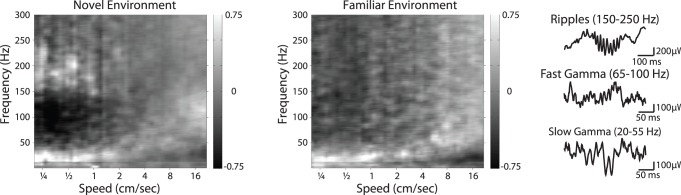
The spectrum of rhythmic local field potential activity in area CA1 is smoothly modulated by behavior. Example spectrograms across speeds. Power in each frequency is shown as a z-score relative to the mean power across speeds. **(left)** Spectrogram calculated in the first exposure to a novel environment. **(middle)** Spectrogram calculated after the environment has become familiar (epoch 12). **(right)** Three known and physiologically relevant rhythms, the ripple oscillation (150–250 Hz), fast gamma (65–100 Hz), and slow gamma (25–55 Hz).

#### Gamma Oscillation Analyses

**Figure 3:**
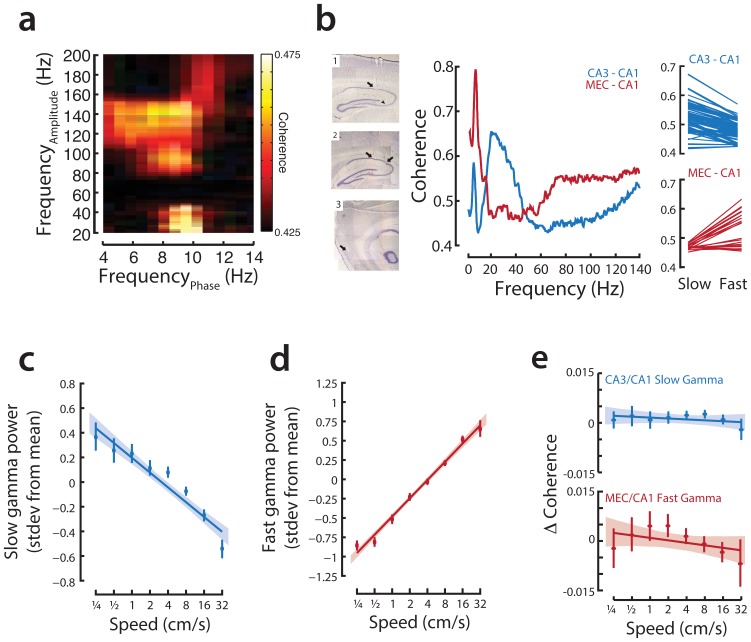
Slow and fast gamma oscillations in CA1 are modulated by speed. **a,** There are two distinct gamma bands in CA1. Shown is an example cross-frequency coherence plot from a novel run session showing that gamma power (y-axis) is modulated by the theta phase (x-axis). **b,** CA1 is more coherent with CA3 in slow gamma range and more coherent with layer 3 of the medial entorhinal cortex in fast gamma range. Coronal and sagittal sections show electrolytic lesions in recording sites from CA3-CA1 recordings (1) and CA1-MEC recordings (2 and 3). Coherence plots for a representative pair of CA3-CA1 (blue) and MEC-CA1 (red) recordings. There is a pronounced peak in the slow gamma range (25–55 Hz) of CA3-CA1 coherence, and a noticeable increase in the fast gamma range (∼70–130 Hz) of MEC-CA1 coherence. Slow gamma coherence was greater than fast gamma coherence for 80 out of 85 pairs of CA3-CA1 recordings (z-test for proportions, p<10^−5^; n = 5 animals), while fast gamma coherence was greater than slow gamma coherence for 19 out of 22 pairs of MEC-CA1 recordings (z-test for proportions, p<10^−5^; n = 3 animals). **c,** Population data showing normalized power of slow gamma vs. speed. Points represent binned means with standard error using logarithmically spaced bin centers; line represents linear regression of underlying data with 95% confidence intervals. **d,** Population data showing normalized power of fast gamma oscillations vs. speed. Points and line as in (**c**). **e,** There are very small changes in coherence as a function of behavioral state. **(top)** Average change in coherence between CA3 and CA1 in the slow gamma frequency band (n = 4 animals) as a function of speed (average slow gamma coherence between CA3 and CA1 = 0.51). Coherence was measured for all tetrode pairs. For each pair, the deviation from the mean value in each 500 ms time window was calculated. Each time window was associated with the appropriate speed. Shown are the regression of speed and change in coherence with 95% C.I. and binned histograms as in (**c**). Change in coherence vs. speed is not significantly different from zero (bootstrap test). **(bottom)** Average change in coherence between MEC and CA1 in the fast gamma frequency band (n = 3 animals, average mean fast gamma coherence between MEC and CA1 = 0.49). Change in coherence vs. speed is not significantly different from zero (bootstrap test).

Previous studies had demonstrated the presence of two distinct gamma bands in CA1, a slow gamma band where CA3 and CA1 oscillate coherently and a fast gamma band where the entorhinal cortex and CA1 oscillate coherently [19]. We first replicated the analyses in that previous paper. As in Colgin et. al. [19], we identified slow and fast gamma bands by computing a cross-frequency coherence [19] between gamma frequencies and the theta rhythm (Figure 3a) and established CA3-CA1 coupling during slow gamma and EC-CA1 coupling during fast gamma (Figure 3b). To investigate how these distinct gamma bands were modulated by speed we computed a normalized power spectrum using 0.5s windows for each tetrode located in the CA1 cell layer. Different recording sites typically produce gamma signals of different absolute power. As a result, combining data across animals requires normalization of the measurements. We therefore computed a z-score for power at each frequency and used this normalized estimate of power for coming across epochs or animals. We then summed the z-scored power across each frequency band and averaged that power across tetrodes. This produced a mean gamma power trace for each 0.5 s of data collection.

For regression analyses we eliminated points with a speed below 1/8 cm/sec to ensure robust linear fits in relation to log(speed). We used a bootstrap resampling method with linear regression, as the distribution of the residuals was non-gaussian, resampling data to ensure equal sampling across four logarithmically spaced speed bins with centers at 1/4, 1, 4, and 16 cm/s. To evaluate the temporal specificity of speed modulation, we measured the Spearman correlation (a non-parametric measure) of normalized gamma power with log(speed) in the concurrent and adjacent 0.5 s time bins. We used a bootstrap method to estimate the mean and 95% confidence interval of the correlation that allowed us to control for different time spent at each movement speed. To test for significant changes in slope over days, we used a permutation test to test against the null hypothesis. To measure the within day differences in gamma power between novel and familiar experiences, we normalized all data to the power spectrum from the familiar session. After binning the data into logarithmically spaced speed bins we used a rank sum test to measure the difference in normalized gamma power between comparable speeds in the novel and familiar track. To measure the within day differences in the depth of modulation between novel and familiar experience we computed 1000 bootstrap estimates of the slope to ensure equal sampling across animals and across different movement speeds.

#### Theta power

To compare whether speed or the strength of the theta rhythm was more predictive, we extracted theta power from our recordings. When available (n = 2), we measured the theta power using recordings in the hippocampal fissure. Alternatively, we used the callosal reference electrodes (n = 5), or if these were not available, electrodes in area CA3 (n = 2). We measured theta power by first 7–9 Hz bandpass filtering (FIR filter, 6, 10 Hz stopband) the recorded LFP. Our measure of theta power was the magnitude of the Hilbert transform of the filtered LFP. For the analysis of the modulation of gamma power by theta power, we used the theta power averaged over the 0.5 s spectral estimation window. For the analysis of the modulation of evoked fPSP slope by theta power, we used the average theta power measured in the second prior to stimulation. To combine theta power across pooled data sets we computed a z-score of theta power across each epoch.

#### Ripple oscillations

Ripple events in CA1 are generally preceded by activation of CA3 neurons, so we also examined ripple oscillations to understand how their amplitude changed with movement speed. We used LFP characteristics and the presence of multiple pyramidal cells to select tetrodes that were located in the CA1 cell layer. We filtered the field potentials recorded from these tetrodes using a 30 tap 150–250 Hz bandpass FIR filter (100, 300 Hz stop band). The magnitude of this filtered signal was then extracted using the Hilbert transform. Ripple events were detected using the criterion that the magnitude of the ripple-band field had to exceed its mean by a threshold of three standard deviations for at least 15 ms. Normalized ripple power was defined as standard deviations above this threshold [24]. The magnitude for each ripple event was defined as the mean across tetrodes for which that event was detected. Note that the ripple magnitude is a normalized measure and thus did not require further normalization to compare across animals. To look at the relationship between movement speed and ripple power, we computed the linear regression between the log of the mean speed during each ripple event and the normalized power of each event. As with gamma power, we eliminated points with a speed below 1/8 cm/sec and used bootstrap resampling and permutation tests for regression analyses. As with gamma power, to measure the within day differences in ripple power between novel and familiar experiences, we normalized all data to the familiar session.

### Measuring Evoked fPSPs

For measuring evoked responses from stimulation of the SC pathway the best measurements are obtained below *stratum pyramidale*, in which a large fPSP can be recorded with a peak amplitude at approximately 8–12 ms. In recordings where multiple tetrodes were available, we chose the tetrode with the largest consistent response. By recording below the cell layer, we attempted to avoid contamination from population spikes. For the silicon probe recordings of stimulation of the SC pathways, we measured the fPSPs from recording sites near the *stratum radiatum* current sink revealed via current-source density analysis. As is typical, we used the slope of the fPSP as our measure. In order to combine across different recording days, we normalized using a multiplicative normalization (as is typical for fPSPs), scaling each measurement by the average across all evoked responses during a given session. For the analysis of the effect of environmental novelty, we normalized fPSP measurements in both sessions by the average from the familiar session. For regression analyses we eliminated points with a speed below 1/8 cm/sec ensure robust linear fits in relation to log(speed).

### Residual Correlations

Our analyses of the LFPs and evoked potentials suggested stronger CA3-CA1 coupling at low speeds. We therefore examined the relationship between CA3 and CA1 spiking as a function of speed. In particular, we asked whether the strength of correlations within CA3-CA1 cell assemblies varied with speed. To do so we computed the residual correlation between pairs of neurons with overlapping place fields. This approach, adapted from Singer et. al. [33] examines the “noise” correlations, or how correlated trial to trial variability is among cell pairs. Residuals were calculated for each neuron as the difference between the expected number of spikes and the actual number of spikes recorded in 125 ms bins. Spikes that occurred during ripples were excluded. To determine the expected number of spikes we computed the expected firing rate in 33 ms bins based on the animal’s location in the track and the linearized place field. We then integrated that rate across each 125 ms time bin and calculated the residuals in each time bin as the difference between the expected number of spikes and the actual number of spikes recorded. We assigned each 125 ms bin to one of four logarithmically spaced speed bins and computed the correlation between residuals of cell pairs. Correlations were only computed if there were at least 2.5 s of data, e.g., ≥20 bins in which both cells’ expected firing rate was >0 Hz. These analyses were repeated for CA1-CA1 cell pairs to examine the effect of speed on the correlations of hippocampal spiking output.

## Results

### Continuous Changes in Hippocampal Local Field Potential during Exploration

We investigated the influence of CA3 on CA1 while rats learned a hippocampally-dependent spatial alternation task in an initially novel W-shaped maze (Figure 1a) [26], [30]. Both the maze and the available distal cues were entirely novel when animals were first exposed to the environment. We used head-mounted light-emitting diodes to track head movement in the plane of the maze and estimated two-dimensional movement speed by smoothing the temporal derivative of position with a Gaussian with a standard deviation of 0.5 seconds (see Methods). As expected, animals moved at a variety of speeds reflecting different types of movement (Figure 1b) and initially spent more time exploring the environment. A signature of this exploration was an increase in periods when the rats moved at intermediate two-dimensional speeds (Figure 1c). Intermediate speeds were only weakly correlated with speed along the axis of the track (Figure 1d; no significant changes in correlations with experience). This indicates that the rats did not simply move towards goal locations more slowly in novel environments; rather, movement at intermediate speeds reflected exploratory behavior, as opposed to linear, goal directed motion. As the environment became more familiar, the distribution of movement speed shifted rightwards as animals spent more of their time running quickly between goal locations (Figure 1c).

We then asked how these changes in movement speed manifested as differences in LFP power at different frequencies. We calculated spectra in 0.5 s bins as a function of the associated movement speeds and, consistent with previous reports of continuous changes in gamma frequency with speed [20], we observed clear changes in LFP power with speed. We then sought to relate these changes to information flow in the hippocampal circuit. In particular, we noted that there were changes in power in three physiologically relevant frequency ranges (Figure 2), two associated with CA3 drive: slow gamma (∼20–55 Hz) and ripple (150–250 Hz) oscillations, and one which has been associated with EC drive: fast gamma oscillations (∼65–140 Hz). As seen in this example, with increasing speed in the novel environment there was an apparent decrease in the power of rhythms associated with CA3 input and a concurrent increase in the power of fast gamma. The tradeoff between these regimes occurred smoothly as a function of speed in both novel and familiar environments but the relationship between speed and CA3 associated rhythms appeared stronger during novelty (Figure 2). To quantify these observations, we investigated the modulation of each frequency band separately.

### Speed Modulates Gamma Power during Learning

Previous work had demonstrated overall increases in gamma frequency with movement speed [20], but did not examine these changes in the context of specific bands of gamma associated with CA3 and EC coherence with CA1. In particular, Colgin et. al. [19] demonstrated that CA3 and CA1 are most coherent at slow gamma frequencies (∼20–55 Hz) while EC and CA1 are most coherent at fast gamma frequencies (∼65–140 Hz) and that fast gamma occupies a preferred phase of the theta oscillation [34]. These findings have been interpreted to indicate that periods of increased slow gamma power in CA1 are indicative of greater CA3 drive, while increases in fast gamma power are indicative of greater EC drive [4], [19]. We replicated these findings in our data. We observed both slow and fast gamma oscillations in CA1 (Figure 3a). The presence of slow and fast gamma in CA1 was consistent with differential CA3 and EC drive of CA1∶80 of 85 CA3-CA1 electrode pairs showed higher coherence in slow, as compared to fast gamma (p<10^−10^; Figure 3b) while 19 of 22 CA1-EC electrode pairs showed higher coherence in fast, as compared to slow gamma (p<10^−5^). We also noted that these proportions could be underestimates, as our recording sites in the CA1 cell layer are likely not optimal for measuring slow and fast gamma power. Next we asked how gamma power in these bands varied with movement speed.

We first focused on recordings from the first novel exposure to a W-track and computed a z-scored gamma power in slow and fast bands for each animal to permit pooling of data across animals. We found that the normalized power of slow gamma was largest when the animal was still and decreased smoothly with the log of movement speed (Figure 3c; bootstrap linear regression, normalized slow gamma power vs. log(speed); p<10^−5^). Further, when we binned speed logarithmically, speeds of 1, 4, and 16 cm/sec were all associated with significantly different levels of slow gamma power from each other (Kruskal-Wallis ANOVA, post-hoc tests, p<0.01). In sharp contrast, the power of fast gamma oscillations continuously increased with increasing speed (Figure 3d; bootstrap linear regression, normalized fast gamma power vs. log(speed); p<10^−5^). Fast gamma power was significantly different between all non-adjacent speed bins (Kruskal-Wallis ANOVA, post-hoc tests, p<10^−5^). These findings are consistent with previous reports of positive correlation between high gamma power and speed [35] and slightly higher average speeds associated with fast as compared to slow gamma [19]. Note, however, that the slow gamma band we identify through coherence differs from previous reports of slow gamma/beta in mice [35]–[37], which may explain the previous report of increases in slow gamma with speed in mouse [35].

We previously showed that CA3-CA1 slow gamma power and coherence both increase during SWR events [38], which generally, but not always, reflect strong CA3 input to CA1 [6], [39]. We therefore asked whether changes in gamma coherence accompanied the changes in slow and fast gamma power as a function of speed. Interestingly, we found no evidence for any overall changes in either CA3-CA1 slow gamma or EC-CA1 fast gamma coherence (Figure 3e). This indicates that the mechanisms that drive the changes in CA1 gamma power as a function of movement speed do not alter the overall coherence in gamma between CA1 and its two main inputs.

**Figure 4:**
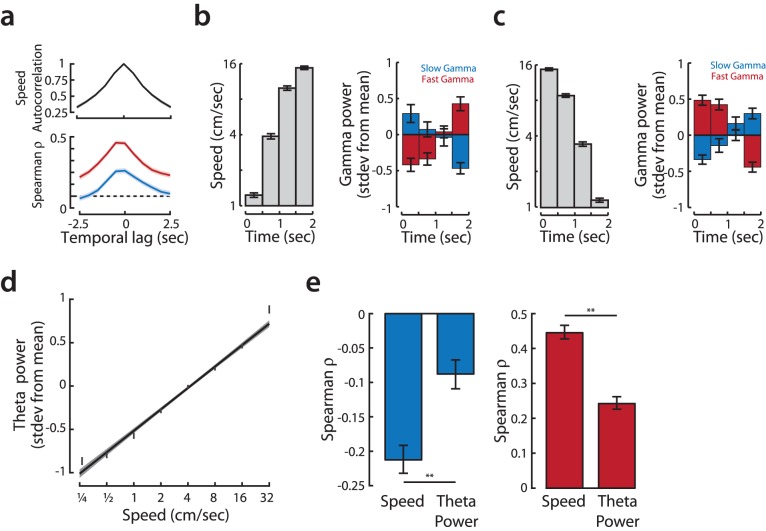
Modulation of gamma oscillations by speed in CA1 is rapid. **a, (top)** The autocorrelation of speed (shown with bootstrap 95% confidence interval) during the first exposure shows a rapid fall-off of on the timescale of ∼1 second. **(bottom)** Both fast and slow gamma power is most modulated by the animal’s speed measured within a second of the gamma power estimate, implying a rapid timescale of modulation. Shown are the Spearman correlation and bootstrap 95% confidence interval of fast (red) and slow (blue) gamma power with log(speed) for offsets in speed measurement ranging from −2.5 to 2.5 s relative to the 0.5 s window used to estimate gamma power. **b,** Rapid and opposing changes in the power of slow and fast gamma are apparent in 2 second windows over which rats’ speed increase from less than 2 cm/s to more than 10 cm/s. **(left)** Increasing mean speed (with standard errors) in 0.5 s windows corresponding to the isolated increasing speed incidents. **(right)** Mean slow gamma power (blue bars with standard errors) decreases significantly, while fast gamma simultaneously increases significantly (red bars) over the course of the two-second increasing speed events. **c,** Same analysis as (**b**) but for 2 s windows when speed decreased from more than 10 cm/s to less than 2 cm/s. Periods of decreasing speed are marked by an increase in slow gamma power (blue bars) and a concomitant decrease in fast gamma power (red bars). **d,** Correlation between theta power and log(speed). The graph shows population data of normalized power of theta oscillation (7–9 Hz) vs. speed, both measured over 0.5 s windows. Points represent binned means with standard error; line shows linear regression of underlying data with 95% confidence intervals. **e,** Speed is a better predictor of fast and slow gamma power than theta power. Comparison of Spearman correlation log(speed) or theta power with fast gamma power (red) and slow gamma power (blue). Depicted are correlations with bootstrap 95% confidence intervals. **p<0.01.

These results and previous findings establish that gamma power in the slow and fast bands changes with speed, but the timescale of these changes had not been investigated. We therefore asked if the modulation of slow and fast gamma power by speed reflect moment-by-moment changes in behavior. If so, then the timescale of changes in gamma power should match the timescale of changes in movement speed. We first calculated the autocorrelations of movement speed and found that speed changes rapidly from second to second (Figure 4a, top). We then computed the cross-correlation between gamma power and speed. If gamma power reflected extended timescale changes in behavior (i.e., “moving” and “still”), then the cross correlation would fall off gradually. In contrast, we find that the correlation between speed and gamma power is largest when the speed and gamma power are measured at the same time (lag = 0), and decreases rapidly with increasing lags (Figure 4a, bottom; correlation decreases below 95% bootstrap C.I. for offsets >0.5 s). The similarity between the timescales of change in behavior and in cross correlation between gamma and speed shows that there is a strong coupling between moment-by-moment movement and the power of gamma oscillations in CA1 on the timescale of a second.

We found the same pattern of rapid change in gamma power when we examined periods of acceleration or deceleration. We isolated two-second segments of behavior in which the rat was rapidly changing speed, either increasing from less than 2 cm/s to more than 10 cm/s or decreasing from more than 10 cm/s to less than 2 cm/s. We then examined the power of fast and slow gamma during these events. When rats accelerated slow gamma power decreased and fast gamma power increased (Figure 4b, 87 events in 6 rats, ANOVA, interaction term and post-hoc tests of initial vs. final power, p<10^−5^). Conversely, as rats decelerated, slow gamma power increased and fast gamma power decreased (Figure 4c; 152 events in 6 rats, all comparisons again p<10^−5^). These observations were robust to the duration and speed criteria used to identify acceleration and deceleration events. Thus, rapid changes in speed correspond to rapid alterations in the relative balance between slow and fast gamma. These findings suggest that, in a novel environment, the balance between these regimes, perhaps reflecting the coupling of CA1 with CA3 and EC, changes dynamically and rapidly in relation to behavioral state.

Our focus on the slow and fast gamma bands was motivated by the possibility of relating activity in these bands to CA3 versus EC drive of CA1. Although the lower frequency 7–9 Hz theta rhythm has not been linked to a specific hippocampal pathway, theta strongly modulates hippocampal activity [40]. Further, theta power has been shown to increase with movement speed in familiar environments [41] and theta frequency is reduced in novel environments [22]. These observations led us to ask how theta power changed with movement speed in novel environments. We found that theta power was correlated with speed during the first novel exposure (Figure 4d; R^2^ = 0.17), but the correlation we measured was smaller than previous reports [42] probably due to the novelty of the environment and our inclusion of all behavioral states (as opposed to selecting for high-speed running). We next asked whether theta power or movement speed was a better predictor of slow and fast gamma power during the first exposure to a novel environment. Interestingly, we found that the correlation between speed and slow and fast gamma power was nearly twice that of the correlation between theta and gamma power (Figure 4e, pooled data: ρ_speed_>ρ_theta_, bootstrap p<0.01 for both slow and fast gamma; p<0.05 for 5 of 6 animals individually). These results should be interpreted cautiously, as it is possible that an alternative recording site for theta would have produced a stronger theta signal and better correlations, but it is nonetheless the case that our findings point to movement speed as a strong predictor of hippocampal information processing state.

### Decreasing Modulation of Gamma Power with Increased Familiarity

As an environment becomes more familiar, animals begin to move faster, engaging in more goal directed movement. Activity patterns in CA1 change over this same time period, reflecting the development of stable representations [26], [43], [44]. These changes in behavior and CA1 output as well as the particular importance of area CA3 for rapid learning lead us to ask whether the influence of speed on the slow and fast gamma changed with experience.

**Figure 5:**
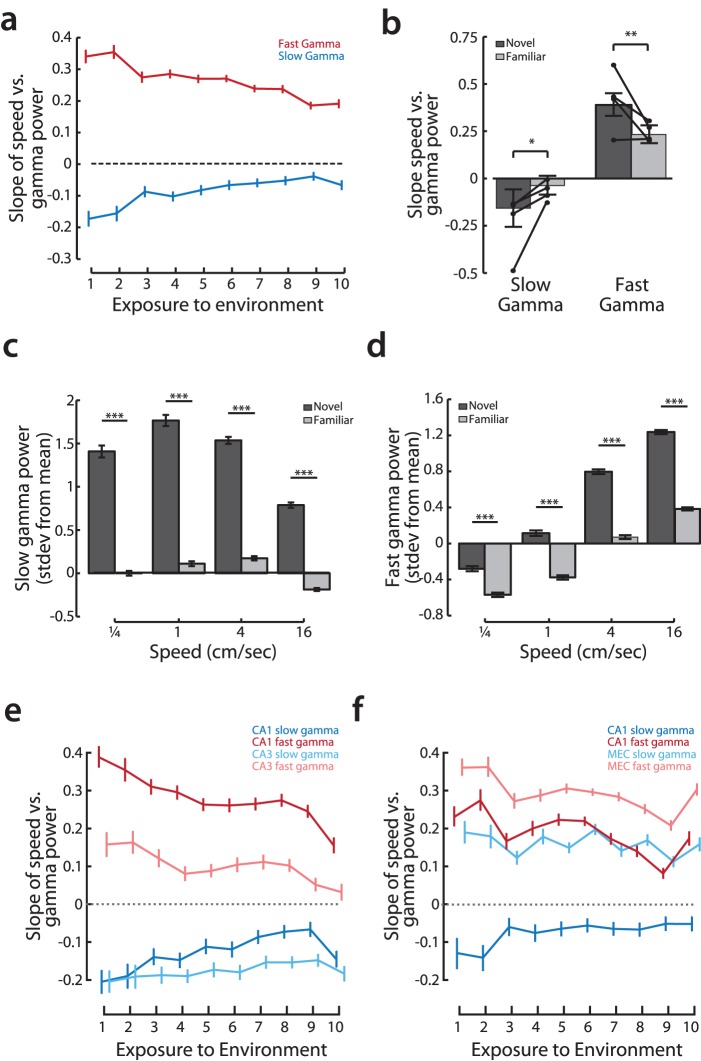
Novelty additionally modulates slow and fast gamma oscillations in CA1. **a,** Slope of the linear regression of slow and fast gamma power vs. speed plotted across exposures to the environment. Points show slope with 95% confidence bounds by bootstrap. **b,** The depth of gamma modulation by speed (more negative slope for slow gamma and more positive slope for fast gamma) is larger in a novel as compared to a familiar environment. Bars show grouped data, means with 95% confidence intervals, thin lines show change in slope for individual animals (bootstrap estimate of slope, novel slope vs. familiar slope; slow gamma p<0.01 for group, p<0.05 for individuals; fast gamma p<0.001 for group, p<0.05 for 3 out of 4 individual animals, one animal n.s.). *p<0.05; **p<0.001 **c–d,** Novelty enhances the depth of modulation as well as the amplitude of gamma oscillations. On days in which a second novel environment was experienced immediately following the first, more familiar environment (n = 4; see methods), the effect of novelty on slow (**c**) and fast (**d**) gamma power could be directly measured. Shown are the mean binned gamma power in the novel (dark gray) and familiar (light gray) environments. Slow and fast gamma power are significantly increased in the novel as compared to the familiar environment for all speeds (rank sum test between novel and familiar session; slow gamma, p<10^−5^ for all speed bins; fast gamma p<10^−5^ for all speed bins). ***p<10^−5^. **e–f,** The power of slow and fast gamma oscillations in the regions which project to area CA1, CA3 and MEC, as a function of speed and novelty. Shown are the slope of gamma power vs log(speed) across exposures with 95% confidence intervals by bootstrap as in (**a**). **e,** Slow gamma is strongly modulated by speed, but not by novelty, in area CA3. CA3 fast gamma is weakly modulated by speed during early exposures (CA1 and CA3 data, n = 4 animals). **f,** Fast gamma is strongly modulated by speed and novelty in EC. Unlike area CA1, slow gamma power increases with speed in EC. (CA1 and EC data, n = 3 animals).

We found that the speed modulation of slow and fast gamma power was strongest during exploration of a novel environment and decreased with increasing familiarity. There was a two-fold decrease in the correlation between speed and gamma power between the first and tenth exposure to an initially novel environment for both slow and fast gamma (Figure 5a; permutation test, exposure vs. slope; slow gamma, p<10^−5^; fast gamma, p<10^−5^). This was observed for both the group data and for each individual animal (permutation test, exposure vs. slope; p<0.01). To disambiguate novelty and task learning and to permit within-dataset comparisons, we took advantage of the second novel environment the animals experienced (see Methods). We compared the slope of the speed versus gamma power relationships across novel and familiar experiences on the same day.

Novelty was associated with greater modulation of both slow and fast gamma as a function of speed (Figure 5b) and with an overall increase in both slow and fast gamma power as compared to that seen on the more familiar track (Figure 5c–d). We also noted that while the speed modulation of fast gamma was evident for both the novel and the familiar environments (Figure 5d), the speed modulation of slow gamma was less evident in the familiar environment. Thus, while there was a general decrease in slow gamma power from lower speeds to the high 16 cm/sec movement speed in novel environments, this modulation was not evident in the familiar environment, consistent with the slope of the gamma versus speed relationship being close to zero for experiences in these familiar places (Figure 5a). Further, the weaker relationship between gamma power and speed in familiar environments as well as the spectral proximity of the first harmonic of the theta oscillation may explain why a recent study in mice exploring a familiar environment reports an increase (rather than decrease) in the power of a putative slow gamma band with speed [35].

We then asked whether these changes in power were also present in CA3 and EC. We found that slow gamma power in CA3 was strongly speed-modulated irrespective of environmental novelty (Figure 5e), suggesting a novelty-related gating of CA3 slow gamma propagation to CA1. In contrast, we found that slow gamma power in EC increased with speed (Figure 5f), confirming that slow gamma in CA1 more closely reflects CA3 drive. There was also a strong speed-modulation of fast gamma in EC, and to a lesser extent in CA3. The strength of this modulation decreased somewhat with experience. Therefore, while speed modulation is present in the inputs to CA1, CA1 integrates these inputs in a novelty-dependent manner.

Our results show that the smooth shift from slow to fast gamma prevalence in CA1 as animals move more quickly is most pronounced in novel environments. Consequently, as animals explore – corresponding to intermediate movement speeds – an important transitional state in the relative prevalence of CA3-associated and EC-associated gamma oscillations is highlighted. In conjunction with the evidence that slow gamma reflects CA3– CA1 coupling while fast gamma reflects EC – CA1 coupling [19] our results suggests that in contexts in which the hippocampus is strongly engaged, the balance of CA1 inputs from CA3 to EC changes gradually and profoundly as a function of movement speed.

### Smooth Modulation of CA1 Ripple Oscillations

**Figure 6:**
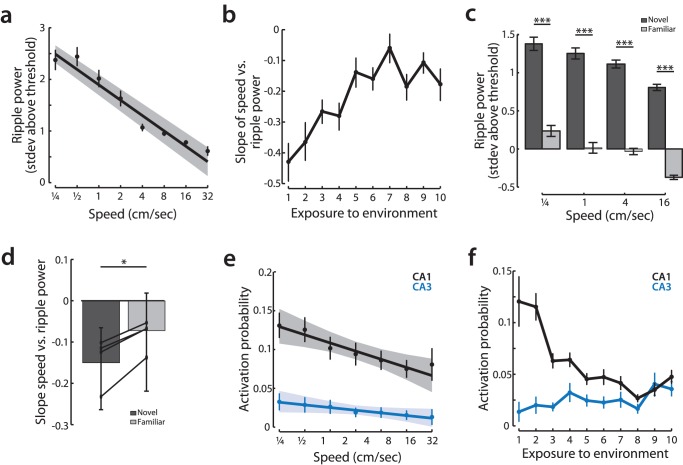
Ripple oscillations and CA1 spiking during ripples reflects the dynamics of CA3 input to CA1. **a,** Normalized ripple power (measured from threshold for detecting ripples) vs. speed. For clarity, normalized ripple power is displayed both as binned averages (binned means and standard error using logarithmically spaced bin centers) and regression to the underlying data (slope with 95% confidence range by bootstrap). **b,** Changes in speed modulation over exposures to the environment. Points represent means +/−95% confidence bounds by bootstrap. **c,** On days in which a second novel environment was experienced immediately following the first, more familiar environment (n = 4; see methods), the effect of novelty on ripple power could be directly measured. Shown are the mean binned ripple power in the novel (dark gray) and familiar (light gray) environments. Ripple power is significantly increased in the novel as compared to the familiar environment for all speeds (rank sum test between novel and familiar session, p<10^−5^ for all speed bins). **d,** The depth of ripple power modulation by speed is larger in a novel as compared to a familiar environment. For all animals where it was possible to measure within day changes, we find that there was a significant increase in the depth of modulation (more negative slope) in the novel as compared to more familiar environment. Bars show grouped data, means with 95% confidence intervals, thin lines show change in slope for individual animals (bootstrap estimate of slope, novel slope vs. familiar slope, p<0.05 for group, p<0.05 for individual animals). *p<0.05; ***p<10^−5^. **e,** Activation probability during each ripple for CA1 and CA3 cells during the first exposure to the environment. For clarity, activation probability is displayed both as binned averages (binned mean and standard error using logarithmically spaced speed bins) and best fit line (linear regression of the underlying data with 95% confidence intervals). **f,** Mean activation probability for CA1 and CA3 neurons across exposures to the environment. Points show mean probability with 95% confidence bounds by bootstrap.

We then asked whether ripple oscillations in CA1 also showed a smooth modulation as a function of movement speed. Ripple oscillations generally reflect synchronized CA3 input [6], although lower-frequency oscillations can still be seen in the absence of CA3 input [39]. Ripple oscillations are also associated with the replay of previously stored memories [12], [13], [45], and this replay involves the coordinated reactivation of CA3 and CA1 neurons [38]. Just as for the CA3-related slow gamma oscillation, we found that during the first exposure to a novel environment, the power of ripple oscillations was largest when the animal was still and decreased smoothly with speed (Figure 6a; bootstrap linear regression, normalized ripple power vs. log(speed); p<10^−5^). We also found that the power of ripple oscillations was most strongly modulated as a function of speed in a novel environment and decreased as the environment became more familiar. There was a three-fold change in slope between the first and tenth exposure to an initially novel environment (Figure 6b; permutation test, exposure vs. slope; p<10^−5^). The enhancement of speed modulation in a novel environment was present in both the group data, for each individual animal, and for within day comparisons (permutation test, exposure vs. slope, p<10^−5^; Figure 6c). Furthermore, as expected [24], novelty was associated with an overall increase in ripple power (Figure 6d).

Our findings for slow gamma suggested that CA3 output to CA1 is gated as a function of speed. We therefore asked whether CA3 and CA1 spiking during ripples was consistent with a gating of CA3 input to CA1 as a function of movement speed. We found that CA1 neurons were most likely to fire during ripples that occurred at slow speeds and became progressively less likely to fire during ripples that occurred at faster speeds (Figure 6e; bootstrap linear regression, activation probability vs. log(speed), p<10^−5^). In contrast, while neurons in CA3 were active during ripples detected in CA1, we found no significant correlation between movement speed and activation probability for CA3 neurons (Figure 6e; bootstrap linear regression, activation probability vs. log(speed); p>0.1). Finally, we found that specifically in CA1, activation probability during ripples was largest during novel experiences and decreased as the environment became more familiar. In contrast the activation probability in CA3 remained stable across time (Figure 6f; permutation set, exposure vs. slope; CA1 p<10^−5^; CA3 p>0.1).

Thus, in a novel environment, rhythms in CA1 associated with CA3 input, both ripple and slow gamma power, are strongly modulated as a function of speed. These results suggest that CA3 input to CA1 is gated as a function of movement speed across the full dynamic range of exploratory behavior. Furthermore, these results suggest that modulation of CA3 input could explain previous observations of greater memory reactivation during and after new experiences [24], [25]. Interestingly, the activity of neurons within CA3 during ripple oscillations showed no modulation by either speed or novelty. This is particularly surprising given that this population activity in CA3 is thought to initiate ripple oscillations [6], [7], [39] and suggests that CA3 activity is relatively constant across speed but that the impact of CA3 output on CA1 changes smoothly with movement speed.

### Smooth Modulation of SC Pathway Strength

The possibility of smooth modulation of the effect of CA3 output on CA1 led us to measure the strength of the SC pathway from CA3 to CA1 as a function of speed. The SC pathway is weaker in moving as compared to still states [16], [17]. However, it is unknown how the strength of the SC pathway varies on a moment-by-moment basis during behavior. To determine whether speed-related changes in the strength of the SC pathway were sufficient to produce the physiological effects we observe, we measured evoked field responses to electrical stimulation of the SC pathway.

**Figure 7:**
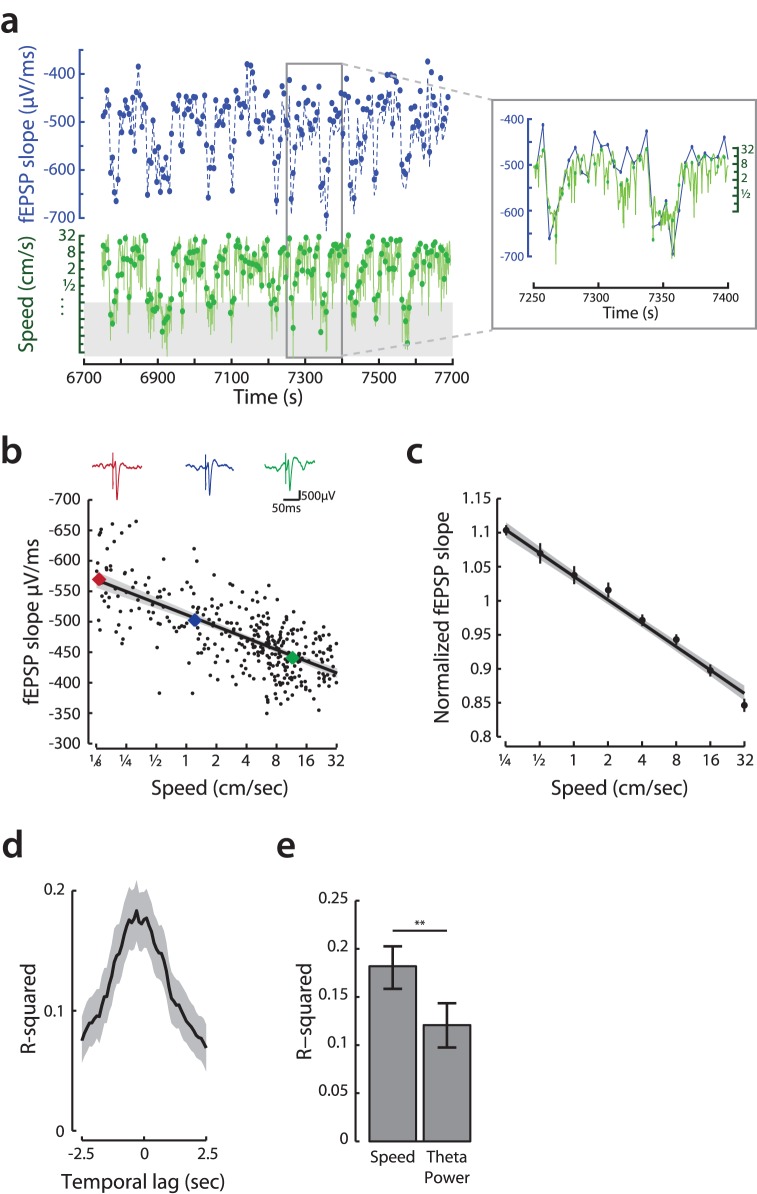
Rapid modulation of the Schaffer collateral pathway as a function of movement speed. **a,** In this study we combined measurements of fPSPs recorded in 4 animals and a total of 31 behavioral epochs. **a,** There is considerable variation in measured fPSP slopes as the rat behaves. In an example epoch (same animal, and one of the two epochs aggregated in Figure 5), the inverse relationship between the slopes of evoked fPSPs (blue dots) and movement speed (green with measurement times denoted with dots) can be observed. Note that the speed axis is logarithmic for similarity with our analyses and that the larger fPSP slopes are at the bottom of the axis. Furthermore, note the times when speeds went below 1/8 cm/s (marked in gray) are excluded from analyses. **b,** Modulation of EPSPs evoked in area CA1 by stimulation of the SC pathway as a function of speed. Colored points represent the measured slopes of the individual fPSP example shown at top. The regression of fPSP slope against log(speed) is shown with a 95% confidence interval estimated by bootstrap. **c,** The modulation of the SC pathway by speed is seen in fPSP measurements normalized and pooled across animals/recording sessions. Normalized measurements are displayed both as binned averages (binned mean and standard error using bin centers on horizontal axis) and a regression line with bootstrapped 95% confidence interval. **d,** The strength of evoked fPSPs is most strongly related to the speed measured at the time of stimulation. Depicted are mean and bootstrapped 95% confidence interval of the R^2^ for regressions of normalized fPSP size to log(offset speed). **e,** Speed is a better predictor of evoked fPSP size than theta_power. Compared are R^2^ for log(speed) vs fPSP slope and for theta power vs fPSP slope. Error bars show 95% confidence interval estimated by bootstrap. **p<0.001.

We found that the slope of the fPSP, a measure of synaptic strength, showed a remarkable level of variation, by as much as 200%, which was strongly correlated with the animals’ instantaneous movement speed (Figure 7a). The evoked fPSPs in area CA1 decreased smoothly with increasing movement speed, with intermediate speeds corresponding to an intermediate synaptic strength (Figure 7b; linear regression, fPSP slope vs. log(speed); F(328) = 305, p<10^−5^, R^2^ = 0.48). This was true both for individual epochs and when normalized responses were pooled across behavioral epochs and animals (Figure 7c; 31 epochs from 4 animals, linear regression, normalized fPSP slope vs. log(speed); F(2770) = 616, p<10^−5^, R^2^ = 0.18). We found that fPSP slopes were significantly different across all non-adjacent speed bins (Kruskal-Wallis one way ANOVA, post-hoc tests, p’s <0.05), such that speeds of 0.25, 1, 4 and 16 cm/sec were all associated with progressively and significantly weaker SC input to CA1. As for gamma power, speed was most predictive of SC strength at short timescales and this correlation decreased significantly for offsets of more than ±0.75 sec (Figure 7d, bootstrapped 95% C.I.). Furthermore, speed was a consistently better predictor of the strength of the SC pathway than theta (Figure 7e; pooled data, bootstrap p<0.001, rank sum test across epochs, p<0.001, p<0.05 for 29 of 31 epochs individually), although we acknowledge that measurements of theta from other parts of the hippocampal formation could in principle produce different results [42].

Here we note that the slope of the fPSP is frequently used as a measure of synaptic strength [16], [46], [47], but multiple factors can influence fPSP slope in-vivo, and so we considered how changes in factors other than synaptic strength would affect the interpretation of our results. For example, increased fPSP amplitude in the dentate gyrus region can result from increases in temperature associated with exploration [48]. Temperature is not a viable explanation for our results, however, as we found that CA1 fPSP slope was largest when animals were still, when brain temperature would be expected to be lowest. We also considered the possibility that post-synaptic membranes were progressively more depolarized at higher speeds, leading to a decrease in the driving force and thus a decrease in fPSP strength. While depolarization of neurons could lead to small changes in driving force, the magnitude of depolarization observed *in vivo* [49] is too small to account for the large changes we observed as a function of speed. We also reasoned that modulation of the SC pathway was unlikely to be due primarily to SC input onto interneurons based on interneuron morphology and the numbers [50]. Thus, the measured change in fPSP slope is best understood as a change in the strength of the SC pathway.

These data also allow us to distinguish between a a continuous system. If the intermediate pathway strength were a result of measurements that came from a combination of two different states, one where the SC pathway was strong and the other where it was weak, the variance of the estimate of the mean would be higher for intermediate speeds than for slow or fast speeds where only one state was present. In strong contrast to that possibility, there were no significant differences among the variances for the measurement of pathway strength as a function of different movement speeds (variances from slowest bin to fastest: 0.0245, 0.0390, 0.0344, 0.0328, 0.0266, 0.0307, 0.0332, 0.0352, Levene’s test, F = 0.81, p = 0.57), consistent with the example in Figure 7b. These results demonstrate that the strength of the SC pathway is modulated continuously as a function of moment-by-moment alterations in speed, which is ideally suited to contribute to the rapid modulation of ripple and slow gamma power observed in CA1. Thus CA3 input to CA1 varies continuously between the extremes observed during awake stillness (strong) and rapid locomotion (weak).

### Smooth Modulation of Correlated Place Cell Activity

Our results indicate that increasing speed is associated with a decrease in the strength of the CA3 inputs to CA1. Furthermore, as the CA3 input decreases in strength, there is a transition from CA3-associated slow gamma to EC-associated fast gamma and a decrease in the amplitude of CA3-driven ripple oscillations in CA1. These findings suggest that the flow of activity from CA3 to CA1 should manifest most clearly at low speeds both during ripple oscillations, as shown in Figure 6, and outside of ripple oscillations in the context of place field activity.

To examine this possibility we excluded ripple events and examined the structure of correlated activity between CA3 and CA1 place cell pairs (n = 4 animals). We asked how the correlation of residuals [33] between pairs of cells with overlapping place fields varied with speed and novelty. The residual correlation (or “noise correlation”) is a measure of how moment-by-moment variability in firing rate co-varies between cell pairs. CA3-CA1 cell pairs with strong residual correlations are likely to be part of functional cell assemblies [51], whereas cell pairs with small residual correlations fire more independently which may be more informative to downstream targets.

**Figure 8:**
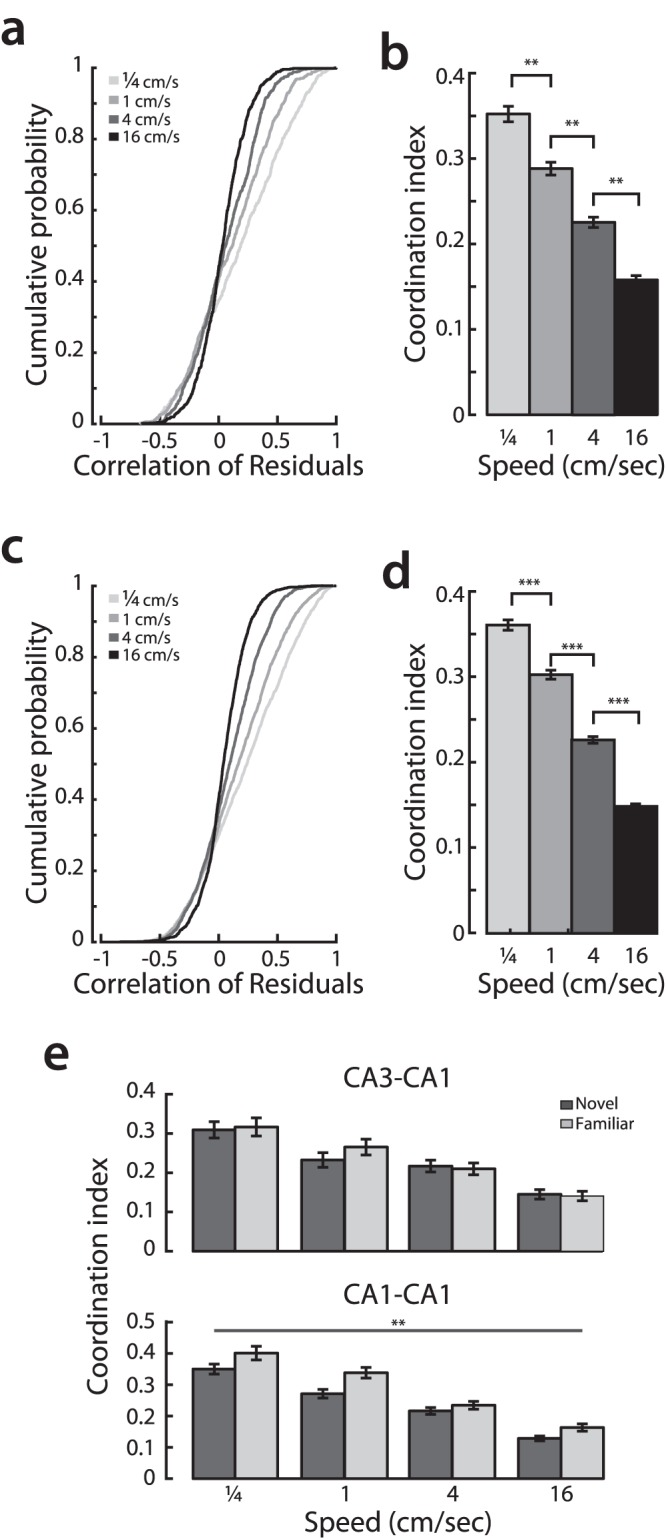
Correlated activity of CA3 and CA1 place cells is modulated by movement speed. **a,** Cumulative distribution of residual correlation of CA3-CA1 cell pairs across speed bins. **b,** Coordination index for CA3-CA1 cell pairs across speeds. **c,** Cumulative distribution of residual correlation of CA1-CA1 cell pairs across speed bins. **d,** Coordination index for CA1-CA1 cell pairs across speeds. **e,** (**top**) The coordination index between CA3 and CA1 place cells is strongly modulated by speed in both familiar (light gray) and novel (dark gray) epochs. However, ANOVA reveals no significant difference between speed modulation in familiar and novel environments. (**bottom**) In contrast, the coordination index of pairs of CA1 cells during the same behavioral epochs shows a significant decrease in the novel environment compared with the familiar one. **p<0.001, ***p<10^−5^.

We found that the distributions of CA3-CA1 residual correlations during the first two days of experience differed markedly across speeds (Figure 8a; KS test all pairs: p<0.0001). To quantify these differences, which consisted of both larger positive and larger negative correlations at low speeds as compared to high speeds, we asked how the absolute value of the residual correlation coefficients, the coordination index, varied across speed. We found that the coordination index was largest at slow speeds and decreased smoothly as animals moved faster (Figure 8b). There was an approximately three-fold decrease in coordinated activity from the slowest to the fastest speed bin, and comparisons across all speed bins were significant (Kruskal-Wallis ANOVA, post-hoc tests, p<0.001). Thus, pairs of co-active neurons had higher residual correlations at slow speeds and became progressively de-correlated as animals moved faster.

We found the same relationships when we examined the residual correlations of CA1 place cell pairs. The correlations of CA1 residuals were strongest at low speeds and decreased significantly as a function of movement speed (Figure 8c–d; Kruskal-Wallis ANOVA, post-hoc tests, p<1×10^−5^). Finally, we asked how novelty modulated the correlation of residuals. We analyzed the data in the subset of animals in which we had isolated CA1 and CA3 place cells on the experimental day in which animals experienced a familiar W-maze followed by a novel W-maze (n = 3, see Methods). Correlating residual firing between CA3 and CA1 place cells, we found the rat’s speed significantly modulated residuals in both novel and familiar environments (Figure 8e, top), but we did not see a significant difference in residual correlations between environments (two-way ANOVA, effect of speed p<0.0001, n.s. effect of environment or interaction term; rank sum test, novel vs. familiar residual correlations for all speeds, p = 0.33). In contrast, analyzing residual firing between the CA1 place cells (Figure 8e, bottom), we found that in a familiar environment, there was an overall increase in the residual correlation as demonstrated by a main effect of novelty (two-way ANOVA, effects of speed and novelty p<0.0001, n.s. interaction term, F = 95.5,17.8, and 0.36 respectively, 1269 total d.f.). This suggests that the learning related increase in residual correlations seen previously [33] may emerge in CA1 independent of CA3.

Taken together, these findings suggest that during exploration of a novel environment, correlated firing in CA1 reflects the rapid associations formed in the CA3 recurrent network when the SC pathway to CA1 is strong at slower speeds. As the environment becomes more familiar, the correlated activity in CA1 may reflect both correlated input from CA3 and associations that have been learned by the network over time. Taken together, these findings indicate that the structure of CA1 spiking changes markedly as a function of movement speed, with the strongly correlated activity seen at low speeds when the influence of the highly recurrent CA3 network is greatest giving way to more independent activity as speed increases.

## Discussion

Our results suggest that as animals behave in a novel environment, the speed of their movement is a simple and parsimonious indicator of the influence of the recurrent CA3 network on CA1. We found that changes in CA3-associated patterns – slow gamma and ripple power – occur on the same timescale as changes in the underlying strength of the SC pathway, directly measured via the slope of evoked fPSPs. This suggests that speed-dependent modulation of the SC is partially responsible for the changes we observe in CA1 activity. While CA1 activity related to CA3 decreases with increasing speed, we found a concomitant increase in the power of fast gamma oscillations, which have been associated with EC inputs [4], [19]. The output spiking activity of CA1 reflected the smooth speed-dependent dynamics seen in gamma and ripple oscillations. At low speeds where CA3 input dominates, CA1 neurons were much more likely to be activated during ripple events and residual correlations of pairs of CA1 neurons were high. At higher speeds CA1 neurons tended to fire more independently, consistent with a decreasing influence of correlated activity from CA3. In a novel environment, animals spend less time moving quickly and more time at intermediate speeds, effectively increasing the influence of the recurrent CA3 network on CA1. In addition to this behavioral bias, novelty itself increases the influence of CA3 to CA1, particularly at low speeds.

It has been shown that the inputs to CA1 are modulated by gross behavioral state and by the phase of the theta oscillation [22], [23], particularly in novel environments. Our findings suggest that changes in the hippocampal pathways, particularly the CA3-CA1 pathway, modulate the patterns of activity expressed in CA1 at an intermediate timescale – the time scale of behavior. As well-trained rats executing behaviors in familiar environments make abrupt changes between low and high speeds, many previous studies of hippocampal activity have simply established maximum and minimum speed thresholds to differentiate between still and moving states. However, for rats in a novel environment, our findings demonstrate that there are significant differences in the prevalence of CA3 and EC associated patterns in CA1 at speeds that traditionally would have been ignored or combined. These fast alterations in circuit activity suggest that a complete understanding of hippocampal activity and its influence on target structures will require knowledge of the specific recent history of neural activity and ongoing behavior.

We found that CA3 activity patterns remain very stable as environments transition from novel to more familiar, while CA1 patterns change markedly with familiarity. Slow gamma in CA3 remained strongly speed modulated across exposures to the initially novel environment, while slow gamma in CA1 became less speed modulated with experience. Similarly, CA3 cells were equally likely to be active during ripple events that occurred at different speeds, and this minimal modulation as a function of movement speed was stable across successive exposures to the environment. In contrast, CA1 cells were more likely to be activated during ripples at low speeds in novel environments as compared to at higher speeds, and the depth of that speed modulation decreased with experience. These findings complement previous work showing that CA3 establishes representations quickly in new places and then remains stable while CA1 is more dynamic. For example, CA3 place fields shift backwards in new environments but not in familiar ones, while CA1 place fields shift each time the animal is placed back in an environment even if it is highly familiar [52]. Furthermore, CA3 firing rates do not change in new places, while CA1 firing rates double [26]. Similarly, CA3 spatial representations remain stable across exposures to an environment, while CA1 representations do not [53], [54].

These differences in CA3 and CA1 responses indicate that CA1 activity is not simply a reflection of feed-forward input from CA3, and our findings provide new insights into how and when CA3 spiking can influence activity in CA1 and how those changes manifest in correlated activity in CA1. First, the strength of the CA3-CA1 SC pathway appears to play an important role in regulating the prevalence of slow gamma, ripples and correlated spiking in CA1. It has long been known that increasing speed is associated with an increase in the firing rate of place cells in area CA1 [55]. Our findings suggest that this may be a direct consequence of changes in the balance of CA3 and EC input. Second, during initial exposures to a novel environment, CA3-associated slow gamma and ripple oscillations are substantially stronger at low speeds while EC-associated fast gamma is increased at high speeds. This suggests that new experiences result in greater drive to CA1 at all speeds, providing a potential explanation for the higher firing rates seen in CA1 during new experiences [26], [43]. Speed-dependent modulation was less prevalent in familiar environments, perhaps allowing for greater task-related modulation independent from behavior [56].

Behavior in novel environments is complex, and many subtle behavior factors influence hippocampal dynamics [57], [58]. Nonetheless, movement speed tracked using head-mounted diodes provides a simple experimentally measurable parameter that effectively characterizes the coupling between CA3 and CA1 in the context of slow gamma, ripples, evoked potentials and correlated spiking. Low and intermediate values of movement speed were both only weakly correlated with speed along the axis of the track and were more prevalent in novel environments, indicating movement speed in our task captures important elements of behavior associated with learning. Movement speed is a behavioral parameter, however, and thus cannot directly affect the strength of specific hippocampal pathways. Nonetheless, differences in speed captured as much as 50% of the variance in fPSP slope, indicating that the causal factor(s) must be tightly correlated with speed. Further, speed modulation was strongest in novel environments, indicating that the causal factor(s) should be enhanced during learning.

Septal modulation of the hippocampal circuit is a likely candidate. Previous work has shown that the firing rate of neurons recorded in vivo from the medial septum, which sends glutamatergic, GABA-ergic and cholinergic projections to the hippocampus, increases with movement speed [59]. Intriguingly, acetylcholine has been shown in vitro to differentially suppress both the SC and EC layer III input to CA1 in a dose dependent manner, with significantly greater suppression of the SC pathway [60]. Similarly, cholinergic agonists and antagonists have been shown to respectively weaken and strengthen field responses to SC stimulation [61]. Complementary results demonstrated that acyetylcholine can have very rapid effects on activity and plasticity in the hippocampus, and thus could mediate alterations in network state consistent with rapid changes in behavior [62]. In addition, acetylcholine levels are higher in novel, as compared to familiar environments [63], which could drive stronger suppression of CA3 inputs to CA1 at high speeds in novel, as compared to familiar environments. We note, however, that some other factor would be required to explain the overall greater levels of slow gamma in novel, as compared to familiar, environments. Finally, septal modulation is also involved in regulating the hippocampal theta rhythm [64] which changes markedly with novelty and speed [22], [65]. These results argue that septal input could play a central role in regulating the moment-by-moment changes we observed, although many other factors, including changes in rhythmic dynamics that depend on the dynamics of particular interneuron circuits could contribute as well.

Why might coordinated activity in the CA3-CA1 circuit be expressed dynamically? Correlations among neurons can be useful for driving plasticity or expressing specific stored representations, but correlations can also reduce the overall information content of a neural code. Thus, correlations in place cell activity during exploration could signal specific associations related to a place, but the expression of the coordination makes the spatial information itself less robust. In a novel environment, the increased correlated activity seen both during ripples and in the context of place fields is well suited to promote the plastic changes involved in building a representation of the environment. The more de-correlated place field activity present at high speeds provides an accurate and informative representation of the animal’s current location to downstream structures. In more familiar environments, the highly correlated activity seen at low speeds is well suited to support memory retrieval [66]. This notion is consistent with observation that animals tend to slow down at decision points where memory is required [67] and suggests that memory retrieval will be facilitated at lower movement speeds.

We hypothesize that the continuous modulation of the influence of CA3 on CA1 allows the hippocampus to serve different functions at different times. At slow speeds CA3 input is strongest. At these times, we observe prominent ripple oscillations, which are thought to support memory consolidation via replay of previously stored associations [66]. Intriguingly, the correlated place cell activity we observed at slower speeds also reflects the co-activation of neural ensembles on timescales that are thought to be well suited for driving synaptic plasticity in downstream structures. Taken together, these results suggest that when CA3 input is strongest, CA1 activity reflects associations stored in CA3 that may promote learning at the CA3-CA1 synapse and in distributed neo-cortical circuits and enable retrieval in these networks. As animals move more quickly, activity in CA1 shifts from representing primarily stored associations towards faithful representations of location. These changes take place smoothly as behavioral state changes rather than in the all or none fashion suggested by a two-state model. Thus movement speed and novelty are critical variables in understanding the dynamics of the CA3-CA1 pathway and the structure of the hippocampal output.
